# Magnetic resonance imaging findings in low back pain and lower extremity radicular chronic pain

**Published:** 2012-11

**Authors:** Hamid Reza Saeidiborojeni, Elham Shobeiri, Sepehr Saeidiborojeni

**Affiliations:** ^*a*^Department of Neurosurgery, Kermanshah University of Medical Sciences, Kermanshah, Iran.; ^*b*^Department of Radiology, Kermanshah University of Medical Sciences, Kermanshah, Iran.; ^*c*^Tehran University of Medical Student, Tehran, Iran.

## Abstract

**Background:**

Low back pain (LBP) is one of the most prevalent complaints among the people worldwide. According to the medical statistics, about 80 percent of people have at least one episode of LBP during their lifetime cause them to visit a physician for treatment. LBP is the most prevalent cause of work disability and its related leaves among people under 45 years old. Numerous studies have been conducted on the connection between clinical signs, patients’ complaints, the level of lumbar disc herniation, and abnormal findings of magnetic resonance imaging (MRI). In many cases, contradictory views have been reported and even in some cases canal stenosis and herniated disc have been reported in patient without clinical sings. The aim of this study was to analyze MRI findings in patients with LBP and radicular chronic pain (RCP) attending Imam Reza Hospital (Kermanshah, Iran).

**Methods:**

This cross-sectional study was conducted on 200 patients (100 with LBP and 100 with RCP). Following unsuccessful results of medical treatment for 1 to 48 months, the examination results, demographic information (age, sex, job, etc.) and their complaints along with MRI findings were documented and statistically analyzed. Patients with history of lumbar surgery, infective inflammatory diseases, fractures, tumors, etc were excluded from the study. To determine the significant correlation between qualitative and quantitative variables, the Chi-Square test and the independent t-test were respectively performed using the statistical package of SPSS (Version 12) The P-value of less than 0.05 was considered significant.

**Results:**

In the LBP group, patients complained only of posterior and inferior spinal column pain and did not have any pain distribution toward lower limbs or any other signs. However, in the RCP group, in addition to pain distribution towards the lower limbs, they were complaining about muscular myotonia and sensational disorder and 88% also had low back pain. 94 of them were men and 106 were women.106 subjects (80.5%) were urban residents and 39 (19.5%) rural residents. There were 42.5% housewife, 21.5% employee, 15% workers, 9.5% self-employed, and 8.5% were unemployed. In terms of education, 27% were uneducated, 40.5% with incomplete high school diploma, and 32.5% with high school diploma and higher levels of education. 33.5% of patients had the treatment period of one month, while for the rest this period was more than one month to 4 years. Twenty percent did not rest, 52.5% rested up to 3 weeks and 24% rested up to 3 months. In the RCP group, 41% complained of pain in the right leg, 36% in the left leg, and 23% in both legs. However, in the RCP group, 26% had limb paresis, 5% atrophy, 25% some levels of sensational disorders, and 2% had sphincter disorders.

**MRI findings:**

Herniated disc in the form of protrusion and extrusion at L4-L5 and L5-S1 spaces were mainly observed in patients with RCP rather than those with LBP. Twenty-six patients (13%) had spondylolisthesis of which 12 (46.2%) were in LBP group and the other 14 patients (53.8) in RCP group. Forty-eight patients (24%) had canal stenosis of which 11 patients were from LBP group and 37 from RCP group. Of these 48 patients, 11 had partial stenosis and 37 had absolute stenosis that were in the RCP group.

**Conclusions:**

The most prevalent herniated disc spaces were at the levels of L4-L5 (87%) and S1-S2 (65%). This is because that most of the movements occur in the mobile parts of disc surface and the stresses that are exerted on the mentioned part. Generally, a meaningful correlation between existence of canal stenosis at L4-L5 and L5-S1 levels and the existence of pressure effect on nerve in THECAL SAC and lat recess in patients with RCP was seen; and also a meaningful correlation among muscular myotonia, radicular distribution of limbs’ pain along with compressive effects on the nerve in the lat recess and the thecal sac and spinal canal stenosis were observed. A significant correlation between complaints of RCP in lower limbs and muscular myotonia with these findings was seen but in analyzing the distributing pain patterns or muscular myotonia on the base of RCP such a correlation was not seen.

**Keywords:**

Low back pain, Radicular back pain, Magnetic resonance imaging


Volume 4, Suppl. 1 Nov 2012
Publisher: Kermanshah University of Medical Sciences
URL: http://www.jivresearch.org
Copyright: (cc) BY

**Paper No. 37**

### Magnetic resonance imaging findings in low back pain and lower extremity radicular chronic pain

Hamid Reza Saeidiborojeni [a], Elham Shobeiri [b,*], Sepehr Saeidiborojeni [c]

[a] Department of Neurosurgery, Kermanshah University of Medical Sciences, Kermanshah, Iran.
[b] Department of Radiology, Kermanshah University of Medical Sciences, Kermanshah, Iran.
[c] Tehran University of Medical Student, Tehran, Iran.

### Abstract:

**Background:** Low back pain (LBP) is one of the most prevalent complaints among the people worldwide. According to the medical statistics, about 80 percent of people have at least one episode of LBP during their lifetime cause them to visit a physician for treatment. LBP is the most prevalent cause of work disability and its related leaves among people under 45 years old. Numerous studies have been conducted on the connection between clinical signs, patients' complaints, the level of lumbar disc herniation, and abnormal findings of magnetic resonance imaging (MRI). In many cases, contradictory views have been reported and even in some cases canal stenosis and herniated disc have been reported in patient without clinical sings. The aim of this study was to analyze MRI findings in patients with LBP and radicular chronic pain (RCP) attending Imam Reza Hospital (Kermanshah, Iran).

**Methods:** This cross-sectional study was conducted on 200 patients (100 with LBP and 100 with RCP). Following unsuccessful results of medical treatment for 1 to 48 months, the examination results, demographic information (age, sex, job, etc.) and their complaints along with MRI findings were documented and statistically analyzed. Patients with history of lumbar surgery, infective inflammatory diseases, fractures, tumors, etc were excluded from the study. To determine the significant correlation between qualitative and quantitative variables, the Chi-Square test and the independent t-test were respectively performed using the statistical package of SPSS (Version 12) The P-value of less than 0.05 was considered significant.

**Results:** In the LBP group, patients complained only of posterior and inferior spinal column pain and did not have any pain distribution toward lower limbs or any other signs. However, in the RCP group, in addition to pain distribution towards the lower limbs, they were complaining about muscular myotonia and sensational disorder and 88% also had low back pain. 94 of them were men and 106 were women.106 subjects (80.5%) were urban residents and 39 (19.5%) rural residents. There were 42.5% housewife, 21.5% employee, 15% workers, 9.5% self-employed, and 8.5% were unemployed. In terms of education, 27% were uneducated, 40.5% with incomplete high school diploma, and 32.5% with high school diploma and higher levels of education. 33.5% of patients had the treatment period of one month, while for the rest this period was more than one month to 4 years. Twenty percent did not rest, 52.5% rested up to 3 weeks and 24% rested up to 3 months. In the RCP group, 41% complained of pain in the right leg, 36% in the left leg, and 23% in both legs. However, in the RCP group, 26% had limb paresis, 5% atrophy, 25% some levels of sensational disorders, and 2% had sphincter disorders.

**MRI findings:** Herniated disc in the form of protrusion and extrusion at L4-L5 and L5-S1 spaces were mainly observed in patients with RCP rather than those with LBP. Twenty-six patients (13%) had spondylolisthesis of which 12 (46.2%) were in LBP group and the other 14 patients (53.8) in RCP group. Forty-eight patients (24%) had canal stenosis of which 11 patients were from LBP group and 37 from RCP group. Of these 48 patients, 11 had partial stenosis and 37 had absolute stenosis that were in the RCP group.

**Conclusions:** The most prevalent herniated disc spaces were at the levels of L4-L5 (87%) and S1-S2 (65%). This is because that most of the movements occur in the mobile parts of disc surface and the stresses that are exerted on the mentioned part. Generally, a meaningful correlation between existence of canal stenosis at L4-L5 and L5-S1 levels

Injury&Violence

Volume 4, Suppl. 1 Nov 2012
Publisher: Kermanshah University of Medical Sciences
URL: http://www.jivresearch.org
Copyright: (cc) BY

and the existence of pressure effect on nerve in THECAL SAC and lat recess in patients with RCP was seen; and also a meaningful correlation among muscular myotonia, radicular distribution of limbs' pain along with compressive effects on the nerve in the lat recess and the thecal sac and spinal canal stenosis were observed. A significant correlation between complaints of RCP in lower limbs and muscular myotonia with these findings was seen but in analyzing the distributing pain patterns or muscular myotonia on the base of RCP such a correlation was not seen.

### Keywords:

***Low back pain, Radicular back pain, Magnetic resonance imaging***

*** Corresponding Author at:**
***Elham Shobeiri:*** *Department of Radiology, Kermanshah University of Medical Sciences, Kermanshah, Iran, Tel: 09181310050, Email: elhamshobeiri@gmail.com,* ***(Shobeiri E.).***

